# Irisin as a Regulator of Brain Energy Homeostasis: Implications for Age-Related Neurodegenerative Diseases

**DOI:** 10.3390/cells15070603

**Published:** 2026-03-28

**Authors:** Bartosz Osuch, Patrycja Młotkowska, Elżbieta Marciniak, Tomasz Misztal

**Affiliations:** Department of Animal Physiology, The Kielanowski Institute of Animal Physiology and Nutrition, Polish Academy of Sciences, Instytucka 3, 05-110 Jabłonna, Poland; p.mlotkowska@ifzz.pl (P.M.); e.marciniak@ifzz.pl (E.M.)

**Keywords:** irisin, FNDC5, AMPK–PGC-1α, BDNF, mitochondrial homeostasis, mitophagy, neurodegenerative diseases

## Abstract

Aging is associated with disturbances in brain energy metabolism, mitochondrial dysfunction, and increased oxidative stress, all of which increase neuronal vulnerability and contribute to the development of neurodegenerative disorders. Growing evidence indicates that physical exercise exerts neuroprotective effects through the release of exerkines–exercise-induced signaling molecules that mediate communication between peripheral tissues and the brain. Among them, irisin, a proteolytic cleavage product of the membrane protein FNDC5, has emerged as an important mediator of the muscle–brain axis. This review summarizes current knowledge on the molecular mechanisms underlying irisin activity in the central nervous system, with particular emphasis on the AMPK–PGC-1α–FNDC5/BDNF signaling axis, rapid receptor-mediated pathways involving the cAMP/PKA/CREB and ERK/CREB cascades, and the regulation of mitochondrial homeostasis, including biogenesis, dynamics, autophagy, and mitophagy. Experimental studies suggest that irisin may improve neuroplasticity, neuronal survival, mitochondrial function, and reduce oxidative stress, thereby alleviating cognitive deficits in models of aging and neurodegeneration. Although the precise receptor mechanisms and intracellular signaling events remain incompletely understood, accumulating evidence identifies irisin as a promising therapeutic target linking metabolic adaptation with neuroprotection. Further investigation of irisin-dependent pathways may facilitate the development of novel strategies aimed at preserving brain function and delaying the progression of age-related neurodegenerative diseases.

## 1. Introduction

Aging is a natural physiological process and represents the major risk factor for age-related neurodegenerative diseases, among which Parkinson’s disease (PD) and Alzheimer’s disease (AD) are the most prevalent. Together, these disorders account for the vast majority of dementia cases and movement impairments in the elderly population. Owing to their high prevalence, well-characterized pathobiological mechanisms, and significant impact on public health, PD and AD constitute typical examples of diseases in which mechanisms associated with biological aging are particularly evident. This is related to the age-dependent accumulation of cellular damage, progressive decline in the efficiency of DNA repair mechanisms, increased oxidative stress, and gradual impairment of mitochondrial function and homeostatic processes within the nervous system [1]. Aging neurons show reduced adaptive capacity, rendering them more susceptible to toxic insults, disturbances in energy metabolism, and inflammatory processes. In addition, brain aging is associated with impaired neurogenesis, lower levels of neurotrophic factors, as well as phenotypic and functional alterations in microglia that promote a chronic pro-inflammatory state [2]. These changes are progressive and largely irreversible, ultimately leading to a substantial decline in patients’ quality of life. Moreover, increasing life expectancy, particularly in developed countries, places a growing burden on healthcare systems worldwide due to age-related neurodegenerative diseases [3]. Despite intensive research, the complex mechanisms underlying age-associated neurodegeneration remain incompletely understood, and current pharmacological interventions are largely symptomatic, aiming primarily to slow disease progression. Consequently, considerable efforts are directed toward elucidating the biochemical pathways and genetic mechanisms involved in neurodegeneration, with the goal of identifying novel therapeutic strategies that target disease pathogenesis rather than symptoms alone [4].

An important research direction concerning potential therapeutic strategies for neurodegenerative diseases involves approaches that improve brain cellular energy metabolism. The brain is one of the most energy-demanding organs in the human body, consuming approximately 20% of the body’s total energy despite accounting for only about 2% of body mass [5,6]. Hence, both neuronal and glial cells are particularly vulnerable to mitochondrial dysfunction, which leads to impaired ATP production, excessive generation of reactive oxygen species (ROS), and disturbances in calcium homeostasis. Oxidative stress, resulting from an imbalance between high free radical production and insufficient antioxidant defense, contributes to damage of proteins, lipids, and DNA, thereby accelerating neurodegenerative processes. Moreover, impaired mitochondrial function and energy distribution may compromise cellular repair mechanisms and exacerbate the accumulation of toxic protein aggregates characteristic of these diseases [7,8].

In this context, exerkines, biologically active molecules released by various tissues during physical exercise, may represent an important molecular link between metabolic adaptation and central neuroenergetic homeostasis during aging. Among these substances, irisin has attracted particular attention. This exercise-induced peptide may participate in the regulation of brain energy homeostasis as well as neuroprotective processes [9]. The present review summarizes the key signaling pathways and molecular mechanisms underlying the effects of irisin in the central nervous system (CNS), with particular emphasis on their relevance to neuronal function and survival in age-related neurodegenerative diseases. In addition, limitations of the current knowledge, methodological challenges, and future research directions that may clarify the translational potential of irisin as a neuroprotective factor are discussed.

## 2. Irisin

One of the key exerkines with documented effects on the CNS is irisin–a myokine secreted by skeletal muscle. In addition, white adipose tissue may serve as an alternative source of irisin in response to physical exercise, accounting for approximately one-third of the total circulating levels of this exocrine in the body [10]. Moreover, local irisin expression has been detected in other organs, including the brain, testes, liver, pancreas, spleen, heart, and stomach [11,12,13]. This widespread distribution allows irisin to act through endocrine, paracrine, and autocrine signaling pathways, thereby exerting both peripheral and central effects. Accordingly, a growing body of research suggests that irisin may directly modulate neuronal function and processes within the CNS. It has been implicated in the regulation of neurogenesis, synaptic plasticity, and neuronal survival. Studies in animal models have indicated that increasing peripheral irisin levels also elevates its concentrations in the cerebrospinal fluid (CSF) and subsequently leads to changes in gene expression within the brain. These findings have been interpreted as evidence that this myokine can influence the CNS function, potentially by crossing the blood–brain barrier (BBB) or by affecting peripheral-to-central signaling pathways [14,15,16,17]. Irisin also exerts neuroprotective effects by stimulating the expression of brain-derived neurotrophic factor (BDNF), a neurotrophin essential for neuronal differentiation, survival, learning, and memory [18]. Moreover, irisin may modulate signaling pathways associated with neuroinflammation and oxidative stress, which is particularly relevant for the prevention and slowing of neurodegenerative disorders such as AD and PD [19,20]. Inflammation induces time-dependent alterations in the expression of pro-inflammatory cytokines and their receptors in many neuroendocrine regulatory axes [21], which may affect systemic and central metabolic homeostasis and alter mitochondrial and neurotrophic regulatory mechanisms.

Despite increasing interest in irisin as a signaling mediator in the muscle–brain axis, the molecular mechanisms underlying its actions in the CNS remain incompletely understood, particularly with respect to specific receptor interactions and downstream intracellular signaling cascades. Expression of fibronectin type III domain-containing protein 5 (FNDC5), the precursor of irisin, has been detected in several regions of both rodent and human brains. In the former, irisin has been observed at particularly high levels in cerebellar Purkinje cells, vestibular nuclei of the medulla oblongata, hippocampus and cerebral cortex [22,23], whereas in primates, robust expression has been reported in the arcuate and ventromedial nuclei of the primate hypothalamus [13]. However, the physiological role of endogenous FNDC5/irisin within the CNS has not yet been fully elucidated. Nevertheless, irisin, a cleavage product of the exercise-induced protein FNDC5, has been shown to exert broad molecular, cellular, and functional effects [24]. For this reason, increasing attention has recently been directed toward its role in regulating brain energy homeostasis and its potential neuroprotective properties. Reduced FNDC5/irisin expression has been reported in mouse models of AD, resulting in impaired long-term potentiation (LTP) and memory deficits, whereas FNDC5 overexpression partially restores memory performance and synaptic plasticity [15]. Additional studies in experimental AD models have demonstrated that peripheral irisin administration improves cognitive function and memory by activating neuroprotective transduction cascades, including the cAMP/PKA/CREB pathway, which is frequently disrupted in this condition [16]. Conversely, AD pathology has been reported to be exacerbated in FNDC5/irisin knockout mice [25].

Irisin acts through both classical metabolic pathways (e.g., AMPK–PGC-1α–FNDC5/BDNF) and rapid receptor-mediated signaling responses involving membrane receptors and kinase cascades. Additionally, irisin can affect mitochondrial dynamics and biogenesis, as well as autophagy and mitophagy, making it an attractive candidate for therapeutic strategies targeting neurodegenerative diseases [26]. However, incomplete knowledge of these processes currently limits the clinical application of irisin as a therapeutic tool for the treatment or prevention of neurodegeneration. Further investigation of the molecular mechanisms of irisin action is needed to identify novel therapeutic targets and to develop effective pharmacological strategies aimed at preserving brain function in the aging population.

## 3. AMPK and the PGC-1α/FNDC5/BDNF Pathway as a Neuroenergetic Axis

Under physiological conditions, physical exercise rapidly increases ATP utilization in skeletal muscle, elevating intracellular ADP and AMP levels and reducing the ATP/AMP ratio [27,28]. These changes are sensed by AMP-activated protein kinase (AMPK), a key signaling molecule that regulates cellular energy status [29], thereby initiating a signaling cascade that links energy demand to intracellular signaling and subsequent metabolic adaptation [30]. AMPK activation initiates a signaling cascade that promotes catabolic processes supporting ATP regeneration, such as glycolysis, fatty acid β-oxidation, autophagy, and mitophagy, while suppressing energy-demanding anabolic processes, including lipid and protein biosynthesis [31,32,33]. AMPK regulates mitochondrial biogenesis partly through phosphorylation and activation of peroxisome proliferator-activated receptor gamma coactivator-1α (PGC-1α) [34]. Increased PGC-1α expression stimulates the production of fibronectin type III domain-containing protein 5 (FNDC5) [35], which is synthesized as a membrane-bound protein in myocytes. Guo et al. [36] demonstrated, at both the mRNA and protein levels, an age-dependent decline in the concentrations of irisin and its precursor FNDC5 in skeletal muscle in aging mice. Phenotypic analyses further revealed exacerbated muscle atrophy, including reduced grip strength, decreased muscle mass, and fiber size, as well as adverse molecular changes in FNDC5-deficient mice compared to wild-type controls. Notably, these effects were reversed by intraperitoneal administration of recombinant irisin to aged mice.

FNDC5 subsequently undergoes proteolytic cleavage, and its extracellular domain, i.e., irisin, is released into the circulation [37]. As an endocrine signaling molecule, irisin mediates communication between skeletal muscle and distant organs. Animal studies have demonstrated that peripheral elevation of irisin levels increases its concentration in the brain, suggesting that it may cross the blood–brain barrier (BBB), although the mechanism and extent of this process remain unclear [25,38]. Within the CNS, irisin exerts potent neuroprotective effects largely through the upregulation of brain-derived neurotrophic factor (BDNF), which supports neuroplasticity and neuronal and mitochondrial metabolism [39,40]. Early studies showed that irisin was able to enhance the proliferation of mouse hippocampal neuronal H19-7 cells [41]. Subsequently, Mohammadi et al. [42] demonstrated a beneficial effect of irisin on synaptic plasticity in the rat hippocampus in vivo, as demonstrated by the stimulation of long-term potentiation (LTP). Accumulating evidence indicates that these and similar central effects of irisin are mainly mediated through the upregulation of BDNF expression [43]. Furthermore, after 10 weeks of physical training in adults at risk of dementia, a positive correlation was observed between serum irisin, BDNF concentrations and cognitive performance [44].

Moderate aerobic exercise has also been shown to activate the PGC-1α/FNDC5/BDNF pathway by stimulating AMPK signaling in the brains of experimental animals, while improving cognitive function [22]. This effect also extends to impairments induced by prior suppression of the PGC-1α/FNDC5/BDNF pathway following intracerebral injection of amyloid β1-42 (Aβ1-42) [45]. These findings support a role for FNDC5/irisin in the prevention of neurodegenerative diseases, particularly AD. Moreover, clinical studies have shown a positive correlation between irisin levels in CSF and both BDNF expression and cognitive performance in AD patients. CSF irisin concentrations have also been positively correlated with Aβ1-42 in CSF [46,47], the lower levels of which are considered a biomarker of pathological amyloid deposition in the brain [48,49]. Collectively, these findings indicate the importance of the PGC-1α/FNDC5/BDNF axis as a potential therapeutic target in neurodegenerative diseases, particularly in the context of aging and metabolic dysfunction [21,22,50]. At the same time, it should be noted that the activation of this axis is neither a single-step nor a self-sufficient process. Mitochondrial biogenesis is a complex, coordinated event that requires stabilization of cellular energy metabolism and may occur secondary to broader signaling networks. Despite compelling evidence supporting the neuroprotective role of the PGC-1α/FNDC5/BDNF pathway, several important research questions remain unresolved. These include the characterization of irisin transport across the BBB, the role of local FNDC5 expression in brain tissue, and the influence of metabolic states and disorders on the efficiency of this signaling axis [51,52,53]. Further studies are required to clarify the interactions between irisin and other neuroplasticity-related pathways and to define more precisely the therapeutic potential of irisin in neurodegenerative diseases [54]. A detailed understanding of these mechanisms may contribute to the development of novel and effective strategies to support brain health during aging and neurodegeneration. Importantly, irisin function is not limited to transcriptional and mitochondrial mechanisms, as studies also indicate its ability to activate rapid, non-genomic signaling pathways that may be critical for short-term regulation of neuronal function [21,50]. These rapid signaling responses may represent an upstream regulatory layer that complements the slower transcriptional program of the AMPK–PGC-1α/FNDC5/BDNF axis and link immediate neuronal signaling events with longer-term metabolic and mitochondrial adaptations [15,22,32,39].

## 4. Rapid Signaling Pathways Activated by Irisin

Irisin released from FNDC5 may also act rapidly and locally within the brain while inducing longer-term adaptive changes. These effects are believed to be mediated by two parallel receptor-dependent signaling cascades: the cAMP/PKA/CREB and ERK/CREB pathways [55,56]. Upon reaching the brain, irisin binds to membrane receptors to trigger intracellular signaling; however, this mechanism has not yet been fully elucidated. Emerging evidence implicates integrin αV/β5 as a main receptor candidate [57,58], a hypothesis initially proposed by Kim et al. [59], who identified integrin αV/β5 heterodimers as key protein complexes transducing irisin signaling in murine adipocytes and osteocytes. Although subsequent studies have extensively investigated irisin activity, particularly with respect to intracellular signaling pathways [60], it remains unclear whether this receptor system represents the sole mechanism mediating irisin signaling within the central nervous system [61]. Activation of integrin receptor may initiate secondary transduction cascades, including changes in cyclic adenosine 3′,5′-monophosphate (cAMP) levels and kinase-dependent pathways; however, the precise sequence of molecular events has not yet been fully characterized.

This process may increase concentrations of intracellular cAMP, which binds to the regulatory subunits of protein kinase A (PKA) and induces conformational changes that release the active catalytic subunits [62]. These PKA subunits subsequently translocate to the nucleus, where they phosphorylate various proteins, including the transcription factor CREB (cAMP response element-binding protein) [63], which plays a well-established role in neuronal plasticity and long-term memory formation in the brain [15,64,65]. Phosphorylated CREB recruits the transcriptional coactivator CBP (CREB-binding protein), which acts as a molecular bridge between activated CREB and the transcriptional machinery and initiates transcription of genes containing cAMP response elements (CREs), such as BDNF, c-fos, and NR4A1 [66,67,68]. Additionally, binding of irisin to the putative integrin αV/β5 receptor may also induce the mitogen-activated protein kinase (MAPK) cascade [69], particularly the extracellular signal-regulated kinase ½ (ERK1/2) pathway, which promotes CREB phosphorylation [70]. Phosphorylated CREB subsequently binds to the promoter region of the BDNF gene in the nucleus, leading to enhanced BDNF transcription [71]. This process is important not only for neuroplasticity but also for neuronal and mitochondrial metabolism. BDNF may regulate PGC-1α expression, forming a positive feedback loop [22,72], which appears to be relevant for future research. It is also possible that chronic irisin exposure activates transcription factors such as nuclear respiratory factor 1 (NRF1) and nuclear respiratory factor 2 (NRF2), which regulate the expression of mitochondrial protein genes.

In this context, rapid signaling events triggered by irisin, such as CREB phosphorylation via the cAMP/PKA and ERK pathways, may represent early regulatory signals that subsequently impact longer-term transcriptional cues. CREB activation induces the expression of neurotrophic and metabolic genes, including BDNF [15,22,66]. Through this sequence of events, rapid receptor-mediated signaling may initiate a cascade that induces mitochondrial biogenesis and metabolic adaptation. Moreover, AMPK activation may coordinate cellular energy status with mitochondrial quality control by promoting PGC-1α-dependent mitochondrial biogenesis and activating autophagy-related pathways, including ULK1-dependent mitophagy [32,34,73]. Together, these observations support the concept that irisin signaling operates within a complex regulatory network in which rapid receptor-mediated pathways initiate CREB-dependent transcriptional responses, while AMPK signaling coordinates longer-term metabolic adaptations involving mitochondrial biogenesis, remodeling, and mitophagy.

Understanding the interplay between the rapid and long-term effects of irisin, including the mechanisms that mediate the transition from transient signaling responses to sustained transcriptional adaptations, remains one of the key challenges for subsequent investigations. Of particular interest is whether prolonged irisin exposure may modify the neuronal phenotype by reprogramming cellular metabolism and synaptic plasticity.

## 5. Regulation of Mitochondrial Network Dynamics

A key transcriptional target of NRF1/2 is mitochondrial transcription factor A (TFAM), which maintains mitochondrial DNA stability and controls mitochondrial number and function within the cell. TFAM is an important regulator of cellular energy metabolism, as it determines the efficiency of oxidative phosphorylation and ATP production, particularly in tissues with high energy demand such as the brain [74]. Deficiency or dysfunction of TFAM has been associated with mitochondrial abnormalities (e.g., encephalomyopathies), increased susceptibility to oxidative stress [75], as well as aging processes [76] and the pathogenesis of neurodegenerative disorders [77,78]. Thus, the PGC-1α–NRF1/2–TFAM axis is considered a key mechanism underlying mitochondrial biogenesis and neuronal adaptation to fluctuating metabolic demands [22,79]. It should be noted that mitochondrial dynamics and quality control mechanisms differ between neurons and glial cells, which may influence cell-type–specific responses to irisin signaling [50,80]. These coordinated processes collectively determine mitochondrial quality and cellular energy efficiency. Mitochondrial homeostasis can be organized into two complementary regulatory programs: (i) a mitochondrial biogenesis program (PGC-1α → NRF1/2 → TFAM), which promotes mitochondrial expansion and functional capacity, and (ii) a mitochondrial dynamics program (PGC-1α → regulation of fusion and fission proteins), which regulates the structural organization and functional integrity of the mitochondrial network.

In this context, mitochondrial biogenesis, morphology, and function are tightly interconnected and functionally interdependent. Alterations in the expression of mitochondrial biogenesis regulators, such as PGC-1α and TFAM, may influence not only mitochondrial abundance but also their morphology and intracellular organization, which constitute additional determinants of proper neuronal function [81,82]. Under conditions of increased energy demand, mitochondria are thought to undergo fusion to form an interconnected reticular network that supports more efficient ATP production and improves bioenergetic efficiency and functional complementation. In contrast, excessive mitochondrial fragmentation, frequently observed in neurodegenerative conditions, may lead to a reduction in mitochondrial membrane potential [83,84]. The impact of changes in neuronal energy metabolism on mitochondrial network diversity and organelle morphology arising from dynamically regulated mitochondrial processes remains incompletely understood.

**Figure 1:**
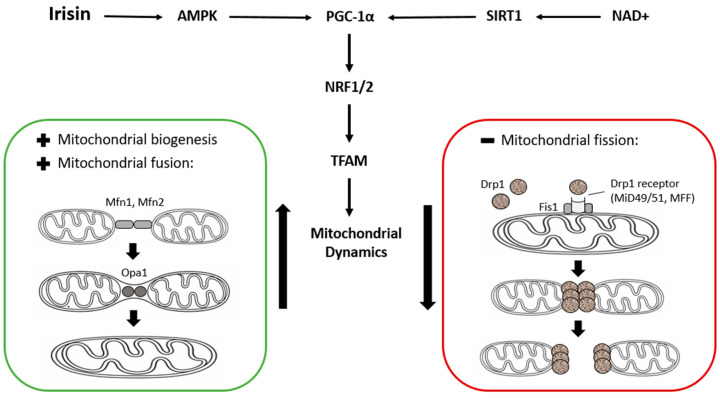
Proposed signaling pathways linking irisin with mitochondrial biogenesis and mitochondrial network dynamics.

The expression of proteins that regulate mitochondrial fusion, i.e., mitofusins (Mfn1 and Mfn2), optic atrophy protein 1 (OPA1), and mitochondrial fission (Drp1, Fis1, Mff, MIEF1, and MiD49/51) may serve as important indicators of the structural and functional state of the mitochondrial network in response to irisin-dependent signaling; however, these relationships require further clarification (Figure 1) [50,85]. Moreover, comprehensive data are lacking regarding how mitochondrial responses are differentially regulated in distinct cell types, brain regions, or metabolic states of the organism [84]. A critical unresolved question in the context of neurodegeneration is whether alterations in mitochondrial dynamics represent a primary cause or a secondary consequence of disrupted energy metabolism and signaling pathways during disease progression [86]. Therefore, future studies should focus on defining the effects of irisin on mitochondrial fusion and fission processes in diverse brain cell populations considering age, sex, metabolic status, and susceptibility to oxidative stress.

Irisin-induced activation of AMPK and increased NAD^+^ availability promote the activation of SIRT1 and the transcriptional coactivator PGC-1α. PGC-1α enhances the expression of nuclear respiratory factors NRF1/2 and mitochondrial transcription factor A (TFAM), thereby regulating mitochondrial biogenesis. These pathways also influence mitochondrial dynamics, including mitochondrial fusion and fission processes. Mitochondrial fusion is regulated mainly by mitofusins (Mfn1, Mfn2) and OPA1, whereas mitochondrial fission is mediated by Drp1 and its mitochondrial receptors (Fis1, MFF, and MiD49/51).

## 6. Autophagy and Mitophagy as Neuroprotection Components

Autophagy and mitophagy can be conceptually organized in a hierarchical manner, where general autophagy represents a broader degradative process and mitophagy constitutes its mitochondria-specific branch. Disruption of the balance between the processes described above, particularly the predominance of mitochondrial fission over fusion, may indicate cellular stress, mitochondrial damage, and early stages of apoptosis [85,86,87]. Thus, preserving proper organization and dynamics of the mitochondrial network not only helps maintain efficient cellular energy production but also plays a crucial role in mechanisms that ensure proper function of individual organelles and survival of the cell as a whole, including mitophagy and autophagy [86,88]. When mitochondrial fragmentation exceeds fusion, the bioelectrical potential of the inner mitochondrial membrane (IMM) decreases, which activates mitophagy, i.e., the selective degradation of damaged mitochondria. This process is tightly regulated by specific effector proteins, PINK1 and Parkin [89,90,91].

PINK1 transiently localizes to the outer mitochondrial membrane, where it functions as a depolarization sensor. In mitochondria with an intact membrane potential, PINK1 is transported from the outer to the inner membrane, where it is cleaved by proteases that have not yet been fully characterized. The remaining fragment is subsequently released into the cytoplasm and degraded proteolytically [89,92,93]. Conversely, PINK1 degradation is inhibited upon loss of mitochondrial membrane potential, leading to the recruitment and activation of the cytosolic E3 ubiquitin ligase Parkin [89,94,95]. Activated Parkin ubiquitinates mitochondrial surface proteins, including Mfn1 and Mfn2, as well as components of the translocase of the outer membrane (TOM) complex [90,96,97]. These ubiquitinated proteins are then recognized by autophagy adaptor proteins, which initiate the engulfment of mitochondria by autophagosomes and their degradation within autophagolysosomes [90,98]. This mechanism enables the cell to eliminate dysfunctional organelles, which limits oxidative stress and prevents apoptosis, a process of particular importance in cells with high energy demands, such as neurons [94,99,100].

In the context of aging and neurodegenerative diseases, the PINK1–Parkin pathway may function both as a biomarker of degeneration and a potential therapeutic target. Maintaining high efficiency of this process is critical, as increased mitochondrial membrane permeability leads to the release of mitochondrial components and degradation products that activate damage-associated molecular patterns (DAMPs) and trigger inflammatory responses [99,101]. Consequently, properly functioning mitophagy may also contribute to the attenuation of chronic inflammatory processes in the aging brain [102,103].

In this framework, the potential role of irisin in modulating autophagy and mitophagy becomes particularly relevant. Although the PINK1–Parkin pathway has been well characterized with respect to the selective elimination of damaged mitochondria, its relationship with irisin activity is largely unexplored [86,89,90]. While preliminary evidence suggests that metabolic factors may influence autophagy and mitophagy, data concerning the direct effects of irisin on the activation of these processes in neuronal cells are currently lacking [32,59,73]. Future studies should determine whether irisin can regulate the PINK1–Parkin pathway and mitochondrial DAMP release and neuroinflammation [101,104]. Identification of such mechanisms could contribute to the development of therapeutic strategies to improve mitochondrial quality control and regulate inflammatory responses in the aging and neurodegenerating brain [14,103].

## 7. AMPK–ULK1–Beclin1–mTOR as an Alternative Autophagy Pathway

Autophagy can be regulated not only through the PINK1–Parkin pathway but also via alternative signaling mechanisms, including the AMPK–ULK1–Beclin1 axis. The PINK1–Parkin pathway is primarily responsible for the recognition and removal of mitochondria with a depolarized inner membrane [89,90]. In contrast, AMPK regulates mitochondrial quality control by activating autophagy-related signaling cascades, including ULK1–Beclin1, in addition to promoting mitochondrial biogenesis through the PGC-1α pathway [105,106,107]. A complementary AMPK–ULK1–Beclin1 pathway can also induce autophagy in response to energy deficiency and metabolic stress, independently of mitochondrial depolarization [73,108,109]. Considering that irisin activates AMPK, it may stimulate not only mitochondrial biogenesis, as discussed above, but also autophagy-related processes [59,110]. This suggests that irisin may primarily influence autophagy through AMPK-dependent pathways, whereas its potential interaction with the PINK1–Parkin mechanism remains to be clarified. Reduced activity of Beclin1 and ULK1 observed in AD correlates with neurodegeneration; therefore, the AMPK–ULK1–Beclin1–(mTOR) axis represents a potential therapeutic target for restoring mitochondrial and cellular homeostasis [111,112,113]. In AD, dysregulation of the AMPK–mTOR axis may impair autophagy activation, causing accumulation of damaged mitochondria, which further aggravates ETC dysfunction and oxidative stress [114,115,116]. Thus, comparative evaluation of the AMPK–ULK1–Beclin1–(mTOR) and PINK1–Parkin pathways in response to irisin would help clarify whether these mechanisms operate independently, hierarchically, or synergistically during metabolic stress. Such analyses could facilitate the identification of the most promising therapeutic targets in neurodegenerative diseases. Despite growing interest in the role of irisin in mitochondrial homeostasis, studies that simultaneously address both autophagy pathways in the context of chronic irisin signaling remain scarce [117]. In this context, the AMPK–ULK1–Beclin1 pathway may represent a complementary or parallel regulatory layer within the broader autophagic response.

## 8. Energy Homeostasis and Neurodegenerative Diseases

From a functional perspective, autophagy represents a fundamental mechanism of neuronal homeostasis and plays a central role in neuroprotection, particularly under conditions of metabolic stress and aging. Autophagic clearance of protein aggregates and dysfunctional organelles is essential for the survival of postmitotic cells, particularly neurons, which have high energy requirements and limited regenerative capacity. These mechanisms play a fundamental role in slowing age-related processes and the prevention and treatment of neurodegenerative diseases [118,119,120]. Impairment of mitochondrial function together with defective autophagic processes disrupts neuronal homeostasis. Age-associated mutations in mitochondrial DNA increase the production of reactive oxygen species (ROS), which disturb calcium homeostasis in neuronal cells and reduces the activity of calcium ATPases [121]. As a result, ATP synthesis declines, oxidative stress increases and uncoupling of the electron transport chain occurs [122,123]. These energetic disturbances contribute to disruption of the respiratory chain in AD, including alterations in complex I [124] and impaired complex IV–linked respiration [125]. A broader overview of mitochondrial dysfunction is provided by D’Alessandro et al. [126]. Although mitochondrial failure is a shared hallmark of AD and PD, disease-specific alterations in affected ETC complexes and upstream molecular events may influence the response to irisin-mediated signaling in a different manner [126,127]. In AD, elevated intracellular calcium levels in neurons may increase tau protein phosphorylation and contribute to pathological processing of amyloid precursor protein (APP), leading to β-amyloid accumulation [124,128]. Progressive mitochondrial dysfunction also reduces ETC efficiency and impairs autophagy and mitophagy, processes required for the removal of damaged cellular structures [129,130]. Reduced activity of these pathways favors further accumulation of toxic proteins and organelles, which intensifies oxidative stress and inflammatory responses [131,132]. Moreover, dysregulation of the AMPK–mTOR balance observed in AD may result in insufficient autophagy activation and inadequate removal of dysfunctional mitochondria [115]. Together, these alterations reflect a combined failure of mitochondrial energy production and autophagic clearance, which underlies the progressive energy deficit observed in neurodegenerative diseases. Therefore, effective therapeutic strategies should simultaneously target mitochondrial function and autophagic processes to restore energy homeostasis. In this framework, irisin may act as a modulatory factor that links energy sensing pathways with mitochondrial adaptation and neuroprotective mechanisms. This conceptual framework links the pathological features of neurodegeneration with potential therapeutic strategies targeting energy metabolism.

In the context of potential therapeutic strategies, increasing attention has been directed toward myomediators such as irisin, which, through activation of AMPK/PGC-1α signaling, simultaneously stimulate mitochondrial biogenesis and mitophagy, thereby helping maintain energy homeostasis [32,73,133]. Emerging evidence also suggests that irisin may reduce oxidative stress and improve ETC function [21,26], demonstrating its potential as a therapeutic target for neurodegenerative diseases [134,135]. From a neuronal perspective, neuroprotection depends on maintaining proper mitochondrial function and balanced processes of fusion, fission, and mitophagy [136,137]. Further research is therefore required to better define the role of irisin in neuronal energy metabolism and to assess its therapeutic applications in age-related neurodegenerative disorders.

## 9. Implications for Future Translational Research and Therapeutic Perspectives

Current experimental evidence indicates that irisin may act as an important regulator of neuronal energy homeostasis. Its activity involves the coordination of mitochondrial biogenesis and dynamics, autophagic processes, oxidative stress responses, and neuroplasticity, largely mediated through the PGC-1α/FNDC5/BDNF signaling axis [21,138]. By potentially crossing the BBB and activating both transcriptional signaling cascades and rapid non-genomic pathways, including the cAMP/PKA/CREB and ERK/CREB axes, irisin may link peripheral metabolic adaptation with central neuroprotective responses [50,139].

Despite encouraging findings from preclinical studies, several aspects of irisin biology remain unresolved, including: (i) the mechanisms regulating irisin transport across the blood–brain barrier and its distribution within the CNS; (ii) the functional significance of local FNDC5 expression in neural tissue; (iii) the identity, specificity, and cell-type–dependent expression of irisin receptors, including the proposed integrin αV/β5 complex; (iv) the context-dependent effects of acute versus chronic irisin signaling under different energetic conditions; (v) the interactions between the PINK1–Parkin and AMPK–ULK1–Beclin1 pathways in irisin-induced selective mitophagy; (vi) the influence of metabolic disorders such as obesity, insulin resistance, and sarcopenia on the processes mediated by the PGC-1α/FNDC5/BDNF axis; and (vii) whether irisin induces sustained mitochondrial and neuroplastic adaptations in vivo [14,21,32,37,50,53,73,140,141].

From a translational perspective, the development of irisin-based therapeutic strategies faces several important challenges [52]. One of these is the limited understanding of the pharmacokinetic and pharmacodynamic profile of irisin, including its stability in circulation and parameters relevant to the development of potential delivery strategies [142]. It is also unclear whether peripheral administration can ensure predictable and biologically meaningful exposure of the CNS to irisin. Although irisin has been detected in cerebrospinal fluid, the mechanisms underlying its transport from the bloodstream to the CSF are not defined [14]. Another issue concerns the possibility of selectively targeting irisin activity to neural tissues. Because irisin participates in inter-organ signaling and exerts numerous metabolic effects outside the CNS, approaches may be required that would increase its availability in the brain while minimizing potential peripheral effects [53]. Translational studies should therefore determine whether improving BBB penetration or more selective activation of signaling pathways in the CNS can increase the efficacy of potential irisin-based interventions [14,53]. Considerable inter-individual variability in endogenous irisin levels is an additional factor complicating potential clinical applications [52]. Available data suggest that circulating irisin concentrations may depend on both physical activity and metabolic status, including obesity and insulin resistance, although the findings are inconsistent. This indicates that individual patient characteristics may influence the baseline state of the muscle–brain axis and should therefore be accounted for when designing future clinical trials and therapeutic interventions [143].

From a broader translational standpoint, modulation of the muscle–brain axis through irisin and its downstream signaling networks represents a promising strategy for targeting bioenergetic and neuroinflammatory disturbances in neurodegenerative diseases [50,138]. Importantly, the therapeutic potential of irisin extends beyond symptomatic relief and may involve improvement of underlying metabolic and mitochondrial dysfunction. A deeper understanding of irisin signaling mechanisms could facilitate the development of targeted pharmacological approaches, including irisin mimetics, particularly in aging populations with limited capacity for regular physical activity [50,134].

## 10. Limitations

Despite growing interest in irisin as a component of the muscle–brain axis and a substantial body of preclinical data suggesting potential neuroprotective effects, the current state of knowledge is insufficient to define the precise role of irisin in regulating brain energy homeostasis. Several key aspects of irisin biology in the brain remain unclear, including how irisin reaches the CNS, its distribution within the brain tissue, and the role of locally expressed FNDC5 in neural cells. In addition, the identity and specificity of irisin receptors in the brain, as well as their cell type- and region-dependent distribution, have not yet been systematically characterized [51,53].

Another important limitation is the incomplete understanding of the long-term consequences of chronic irisin exposure, including its impact on the balance between mitochondrial biogenesis, autophagy, and mitophagy during aging and neurodegenerative diseases [144]. The extent to which findings from animal models can be directly extrapolated to clinical settings, particularly in the presence of concomitant metabolic disorders such as obesity, insulin resistance, or sarcopenia has yet to be established [20,26,145]. These uncertainties are further compounded by potential specific differences between rodent models and humans, particularly in the regulation of FNDC5 expression, circulating irisin levels, and receptor-mediated signaling mechanisms [52]. Finally, it is not yet determined whether modulation of energy-related and mitochondrial pathways by irisin alone is sufficient to alter the course of advanced neurodegenerative processes [54].

The current literature also shows inconsistent findings regarding the relationship between circulating irisin levels and the severity of neurodegenerative pathology [52,146]. Some clinical trials have reported reduced irisin concentrations in patients with Alzheimer’s disease (AD) or Parkinson’s disease (PD), together with associations with cognitive decline or worsening clinical symptoms [46,135]. However, other studies have not confirmed these associations, and the findings remain inconsistent [52,146]. Interpretation of these findings is further complicated by the heterogeneity of clinical populations, differences in disease stage, and the presence of comorbid metabolic disorders such as obesity or insulin resistance [52,142,146,147]. Comparability of results is also limited by methodological differences related to the measurements of irisin levels in biological samples. Previous studies have indicated that results obtained using different analytical methods may vary considerably, and several commonly applied assays have not yet been fully validated for clinical research [52,148]. Consequently, the relationship between irisin levels and the course of neurodegenerative disease progression is still not well understood and requires further well-designed clinical studies.

## Data Availability

No new data were created or analyzed in this study.

## References

[1] Guo J., Huang X., Dou L., Yan M., Shen T., Tang W., Li J. (2022). Aging and aging-related diseases: From molecular mechanisms to interventions and treatments. Signal Transduct. Target. Ther..

[2] Culig L., Chu X., Bohr V.A. (2022). Neurogenesis in aging and age-related neurodegenerative diseases. Ageing Res. Rev..

[3] Wang S., Jiang Y., Yang A., Meng F., Zhang J. (2024). The Expanding Burden of Neurodegenerative Diseases: An Unmet Medical and Social Need. Aging Dis..

[4] Li Z., Zhang Z., Ren Y., Wang Y., Fang J., Yue H., Ma S., Guan F. (2021). Aging and age-related diseases: From mechanisms to therapeutic strategies. Biogerontology.

[5] Padamsey Z., Rochefort N.L. (2023). Paying the brain’s energy bill. Curr. Opin. Neurobiol..

[6] Sanghai N., Tranmer G.K. (2023). Biochemical and Molecular Pathways in Neurodegenerative Diseases: An Integrated View. Cells.

[7] Qin P., Sun Y., Li L. (2024). Mitochondrial dysfunction in chronic neuroinflammatory diseases (Review). Int. J. Mol. Med..

[8] Xu X., Pang Y., Fan X. (2025). Mitochondria in oxidative stress, inflammation and aging: From mechanisms to therapeutic advances. Signal Transduct. Target. Ther..

[9] Avgerinos K.I., Liu J., Dalamaga M. (2023). Could exercise hormone irisin be a therapeutic agent against Parkinson’s and other neurodegenerative diseases?. Metabol. Open..

[10] Roca-Rivada A., Castelao C., Senin L.L., Landrove M.O., Baltar J., Belén Crujeiras A., Seoane L.M., Casanueva F.F., Pardo M. (2013). FNDC5/irisin is not only a myokine but also an adipokine. PLoS ONE.

[11] Aydin S., Kuloglu T., Aydin S., Kalayci M., Yilmaz M., Cakmak T., Albayrak S., Gungor S., Colakoglu N., Ozercan I.H. (2014). A comprehensive immunohistochemical examination of the distribution of the fat-burning protein irisin in biological tissues. Peptides.

[12] Martinez Munoz I.Y., Camarillo Romero E.D.S., Garduno Garcia J.J. (2018). Irisin a novel metabolic biomarker: Present knowledge and future directions. Int. J. Endocrinol..

[13] Wahab F., Khan I.U., Polo I.R., Zubair H., Drummer C., Shahab M., Behr R. (2019). Irisin in the primate hypothalamus and its effect on GnRH in vitro. J. Endocrinol..

[14] Ruan Q., Zhang L., Ruan J., Zhang X., Chen J., Ma C., Yu Z. (2018). Detection and quantitation of irisin in human cerebrospinal fluid by tandem mass spectrometry. Peptides.

[15] Lourenco M.V., Frozza R.L., de Freitas G.B., Zhang H., Kincheski G.C., Ribeiro F.C., Gonçalves R.A., Clarke J.R., Beckman D., Staniszewski A. (2019). Exercise-linked FNDC5/irisin rescues synaptic plasticity and memory defects in Alzheimer’s models. Nat. Med..

[16] Madhu L.N., Somayaji Y., Shetty A.K. (2022). Promise of irisin to attenuate cognitive dysfunction in aging and Alzheimer’s disease. Ageing Res. Rev..

[17] Sadier N.S., Hajjar F.E., Al Sabouri A.A.K., Abou-Abbas L., Siomava N., Almutary A.G., Tambuwala M.M. (2024). Irisin: An unveiled bridge between physical exercise and a healthy brain. Life Sci..

[18] Lourenco M.V., de Freitas G.B., Raony Í., Ferreira S.T., De Felice F.G. (2022). Irisin stimulates protective signaling pathways in rat hippocampal neurons. Front. Cell. Neurosci..

[19] Qi J.Y., Yang L.K., Wang X.S., Wang M., Li X.B., Feng B., Wu Y.M., Zhang K., Liu S.B. (2022). Irisin: A promising treatment for neurodegenerative diseases. Neuroscience.

[20] Paoletti I., Coccurello R. (2024). Irisin: A Multifaceted Hormone Bridging Exercise and Disease Pathophysiology. Int. J. Mol. Sci..

[21] Wójcik M., Zięba D.A., Tomczyk M., Bohenek J., Antushevich H., Krawczyńska A., Herman A.P. (2023). Time-dependent effect of inflammation on the gene expression of pro-inflammatory cytokines and their receptors at the different levels of the somatotropic axis in ewe. J. Anim. Feed Sci..

[22] Wrann C.D., White J.P., Salogiannnis J., Laznik-Bogoslavski D., Wu J., Ma D., Lin J.D., Greenberg M.E., Spiegelman B.M. (2013). Exercise induces hippocampal BDNF through a PGC-1α/FNDC5 pathway. Cell Metab..

[23] Dun S.L., Lyu R.M., Chen Y.H., Chang J.K., Luo J.J., Dun N.J. (2013). Irisin-immunoreactivity in neural and non-neural cells of the rodent. Neuroscience.

[24] Choi J.W., Balakrishnan R. (2026). Aerobic exercise–induced myokine irisin release: A novel strategy to promote neuroprotection and improve cognitive function. Neural Regen. Res..

[25] Islam M.R., Valaris S., Young M.F., Haley E.B., Luo R., Bond S.F., Mazuera S., Kitchen R.R., Caldarone B.J., Bettio L.E.B. (2021). Exercise hormone irisin is a critical regulator of cognitive function. Nat. Metab..

[26] Wen P., Sun Z., Yang D., Li J., Li Z., Zhao M., Wang D., Gou F., Wang J., Dai Y. (2025). Irisin regulates oxidative stress and mitochondrial dysfunction through the UCP2-AMPK pathway in prion diseases. Cell Death Dis..

[27] Huh J.Y., Panagiotou G., Mougios V., Brinkoetter M., Vamvini M.T., Schneider B.E., Mantzoros C.S. (2012). FNDC5 and irisin in humans: I. Predictors of circulating concentrations in serum and plasma and II. mRNA expression and circulating concentrations in response to weight loss and exercise. Metabolism.

[28] Hargreaves M., Spriet L.L. (2020). Skeletal muscle energy metabolism during exercise. Nat. Metab..

[29] Sharma A., Anand S.K., Singh N., Dwivedi U.N., Kakkar P. (2023). AMP-activated protein kinase: An energy sensor and survival mechanism in the reinstatement of metabolic homeostasis. Exp. Cell Res..

[30] Cheng A., Yang Y., Zhou Y., Maharana C., Lu D., Peng W., Liu Y., Wan R., Marosi K., Misiak M. (2016). Mitochondrial SIRT3 Mediates Adaptive Responses of Neurons to Exercise and Metabolic and Excitatory Challenges. Cell Metab..

[31] Hardie D., Ross F., Hawley S. (2012). AMPK: A nutrient and energy sensor that maintains energy homeostasis. Nat. Rev. Mol. Cell Biol..

[32] Herzig S., Shaw R. (2018). AMPK: Guardian of metabolism and mitochondrial homeostasis. Nat. Rev. Mol. Cell Biol..

[33] Fu Y., Yang J., Chen J., Li Y. (2024). The role and molecular mechanisms of copper in regulating animal lipid metabolism. J. Anim. Feed Sci..

[34] Jäger S., Handschin C., St-Pierre J., Spiegelman B.M. (2007). AMP-activated protein kinase (AMPK) action in skeletal muscle via direct phosphorylation of PGC-1α. Proc. Natl. Acad. Sci. USA.

[35] Boström P., Wu J., Jedrychowski M.P., Korde A., Ye L., Lo J.C., Rasbach K.A., Boström E.A., Choi J.H., Long J.Z. (2012). A PGC1-α-dependent myokine that drives brown-fat-like development of white fat and thermogenesis. Nature.

[36] Guo M., Yao J., Li J., Zhang J., Wang D., Zuo H., Zhang Y., Xu B., Zhong Y., Shen F. (2023). Irisin ameliorates age-associated sarcopenia and metabolic dysfunction. J. Cachexia Sarcopenia Muscle.

[37] Wrann C.D. (2015). FNDC5/irisin-their role in the nervous system and as a mediator for beneficial effects of exercise on the brain. Brain Plast..

[38] Kam T.I., Park H., Chou S.C., Van Vranken J.G., Mittenbühler M.J., Kim H., A M., Choi Y.R., Biswas D., Wang J. (2022). Amelioration of pathologic α-synuclein-induced Parkinson’s disease by irisin. Proc. Natl. Acad. Sci. USA.

[39] Marosi K., Mattson M.P. (2014). BDNF mediates adaptive brain and body responses to energetic challenges. Trends Endocrinol. Metab..

[40] Horowitz A.M., Fan X., Bieri G., Smith L.K., Sanchez-Diaz C.I., Schroer A.B., Gontier G., Casaletto K.B., Kramer J.H., Williams K.E. (2020). Blood factors transfer beneficial effects of exercise on neurogenesis and cognition to the aged brain. Science.

[41] Moon H.S., Dincer F., Mantzoros C.S. (2013). Pharmacological concentrations of irisin increase cell proliferation without influencing markers of neurite outgrowth and synaptogenesis in mouse H19-7 hippocampal cell lines. Metabolism.

[42] Mohammadi S., Oryan S., Komaki A., Eidi A., Zarei M. (2019). Effects of intra-dentate gyrus microinjection of myokine irisin on long-term potentiation in male rats. Arq. Neuropsiquiatr..

[43] Natalicchio A., Marrano N., Biondi G., Dipaola L., Spagnuolo R., Cignarelli A., Perrini S., Laviola L., Giorgino F. (2020). Irisin increases the expression of anorexigenic and neurotrophic genes in mouse brain. Diabetes Metab. Res. Rev..

[44] Küster O.C., Laptinskaya D., Fissler P., Schnack C., Zügel M., Nold V., Thurm F., Pleiner S., Karabatsiakis A., von Einem B. (2017). Novel blood-based biomarkers of cognition, stress, and physical or cognitive training in older adults at risk of dementia: Preliminary evidence for a role of BDNF, irisin, and the kynurenine pathway. J. Alzheimer’s Dis..

[45] Azimi M., Gharakhanlou R., Naghdi N., Khodadadi D., Heysieattalab S. (2018). Moderate treadmill exercise ameliorates amyloid-β-induced learning and memory impairment, possibly via increasing AMPK activity and up-regulation of the PGC-1α/FNDC5/BDNF pathway. Peptides.

[46] Lourenco M.V., Ribeiro F.C., Sudo F.K., Drummond C., Assunção N., Vanderborght B., Tovar-Moll F., Mattos P., De Felice F.G., Ferreira S.T. (2020). Cerebrospinal fluid irisin correlates with amyloid-β, BDNF, and cognition in Alzheimer’s disease. Alzheimer’s Dement..

[47] Dicarlo M., Pignataro P., Zecca C., Dell’Abate M.T., Urso D., Gnoni V., Giugno A., Borlizzi F., Zerlotin R., Oranger A. (2024). Irisin Levels in Cerebrospinal Fluid Correlate with Biomarkers and Clinical Dementia Scores in Alzheimer Disease. Ann. Neurol..

[48] Fagan A.M., Mintun M.A., Mach R.H., Lee S.Y., Dence C.S., Shah A.R., LaRossa G.N., Spinner M.L., Klunk W.E., Mathis C.A. (2006). Inverse relation between in vivo amyloid imaging load and cerebrospinal fluid Abeta42 in humans. Ann. Neurol..

[49] Kaiser E., Thomann P.A., Essig M., Schröder J. (2011). β-Amyloid (1-42) Levels in Cerebrospinal Fluid and Cerebral Atrophy in Mild Cognitive Impairment and Alzheimer’s Disease. Dement. Geriatr. Cogn. Dis. Extra.

[50] Muzaffar S., Tyagi A., Pugazhenthi S. (2025). Therapeutic Potential of Irisin in Neurodegenerative Diseases. Int. J. Mol. Sci..

[51] Islam M.R., Young M.F., Wrann C.D., Spiegelman B. (2017). The Role of FNDC5/Irisin in the Nervous System and as a Mediator for Beneficial Effects of Exercise on the Brain. Hormones, Metabolism and the Benefits of Exercise [Internet].

[52] Maak S., Norheim F., Drevon C.A., Erickson H.P. (2021). Progress and Challenges in the Biology of FNDC5 and Irisin. Endocr. Rev..

[53] Zhao R. (2022). Irisin at the crossroads of inter-organ communications: Challenge and implications. Front. Endocrinol..

[54] Peng J., Wu J. (2022). Effects of the FNDC5/Irisin on Elderly Dementia and Cognitive Impairment. Front. Aging Neurosci..

[55] Wang H., Xu J., Lazarovici P., Quirion R., Zheng W. (2018). cAMP Response Element-Binding Protein (CREB): A Possible Signaling Molecule Link in the Pathophysiology of Schizophrenia. Front. Mol. Neurosci..

[56] Kim J., Kaang B.K. (2023). Cyclic AMP response element-binding protein (CREB) transcription factor in astrocytic synaptic communication. Front. Synaptic Neurosci..

[57] A M., Wales T.E., Zhou H., Draga-Coletă S.V., Gorgulla C., Blackmore K.A., Mittenbühler M.J., Kim C.R., Bogoslavski D., Zhang Q. (2023). Irisin acts through its integrin receptor in a two-step process involving extracellular Hsp90α. Mol. Cell.

[58] Wang L., Kulthinee S., Slate-Romano J., Zhao T., Shanmugam H., Dubielecka P.M., Zhang L.X., Qin G., Zhuang S., Chin Y.E. (2023). Inhibition of integrin alpha v/beta 5 mitigates the protective effect induced by irisin in hemorrhage. Exp. Mol. Pathol..

[59] Kim H., Wrann C.D., Jedrychowski M., Vidoni S., Kitase Y., Nagano K., Zhou C., Chou J., Parkman V.A., Novick S.J. (2018). Irisin mediates effects on bone and fat via alphaV integrin receptors. Cell.

[60] Rabiee F., Lachinani L., Ghaedi S., Nasr-Esfahani M.H., Megraw T.L., Ghaedi K. (2020). New insights into the cellular activities of Fndc5/Irisin and its signaling pathways. Cell Biosci..

[61] Szczepkowska A., Bochenek J., Wójcik M., Tomaszewska D. (2023). Effect of caffeine on adenosine and ryanodine receptor gene expression in the hypothalamus, pituitary, and choroid plexus in ewes under basal and LPS challenge conditions. J. Anim. Feed Sci..

[62] Sassone-Corsi P. (2012). The cyclic AMP pathway. Cold Spring Harb. Perspect. Biol..

[63] Chen D., Wang J., Cao J., Zhu G. (2024). cAMP-PKA signaling pathway and anxiety: Where do we go next?. Cell Signal..

[64] Li P., Hu Y., Tong L., Bi X. (2025). High-intensity training on CREB activation for improving brain health: A narrative review of possible molecular talks. Front. Endocrinol..

[65] van Zundert B., Montecino M. (2025). Epigenetics in Learning and Memory. Subcell. Biochem..

[66] Esvald E.E., Tuvikene J., Sirp A., Patil S., Bramham C.R., Timmusk T. (2020). CREB Family Transcription Factors Are Major Mediators of BDNF Transcriptional Autoregulation in Cortical Neurons. J. Neurosci..

[67] Esvald E.E., Tuvikene J., Moistus A., Rannaste K., Kõomägi S., Timmusk T. (2022). Differential Regulation of the BDNF Gene in Cortical and Hippocampal Neurons. J. Neurosci..

[68] Chowdhury M.A.R., An J., Jeong S. (2023). The Pleiotropic Face of CREB Family Transcription Factors. Mol. Cells.

[69] Defilles C., Montero M.P., Lissitzky J.C., Rome S., Siret C., Luis J., André F., Rigot V. (2011). αv integrin processing interferes with the cross-talk between αvβ5/β6 and α2β1 integrins. Biol. Cell..

[70] Lin X., He J., Liu F., Li L., Sun L., Niu L., Xi H., Zhan Y., Liu X., Hu P. (2023). β-adrenergic receptor activation promotes the proliferation of HepG2 cells via the ERK1/2/CREB pathways. Oncol. Lett..

[71] Finkbeiner S., Tavazoie S.F., Maloratsky A., Jacobs K.M., Harris K.M., Greenberg M.E. (1997). CREB: A major mediator of neuronal neurotrophin responses. Neuron.

[72] Yang X., Zhang M., Xie B., Peng Z., Manning J.R., Zimmerman R., Wang Q., Wei A.C., Khalifa M., Reynolds M. (2023). Myocardial brain-derived neurotrophic factor regulates cardiac bioenergetics through the transcription factor Yin Yang 1. Cardiovasc. Res..

[73] Egan D.F., Shackelford D.B., Mihaylova M.M., Gelino S., Kohnz R.A., Mair W., Vasquez D.S., Joshi A., Gwinn D.M., Taylor R. (2011). Phosphorylation of ULK1 (hATG1) by AMP-activated protein kinase connects energy sensing to mitophagy. Science.

[74] Golpich M., Amini E., Mohamed Z., Azman Ali R., Mohamed Ibrahim N., Ahmadiani A. (2017). Mitochondrial Dysfunction and Biogenesis in Neurodegenerative diseases: Pathogenesis and Treatment. CNS Neurosci. Ther..

[75] Woo D.K., Green P.D., Santos J.H., D’Souza A.D., Walther Z., Martin W.D., Christian B.E., Chandel N.S., Shadel G.S. (2012). Mitochondrial genome instability and ROS enhance intestinal tumorigenesis in APC(Min/+) mice. Am. J. Pathol..

[76] Chatterjee D., Das P., Chakrabarti O. (2022). Mitochondrial Epigenetics Regulating Inflammation in Cancer and Aging. Front. Cell Dev. Biol..

[77] Kao L.P., Wolvetang E.J. (2017). Mitochondrial Dysfunction and Mitophagy in Neurodegenerative Diseases. Cell Dev. Biol..

[78] Kang I., Chu C.T., Kaufman B.A. (2018). The mitochondrial transcription factor TFAM in neurodegeneration: Emerging evidence and mechanisms. FEBS Lett..

[79] Scarpulla R.C., Vega R.B., Kelly D.P. (2012). Transcriptional integration of mitochondrial biogenesis. Trends Endocrinol. Metab..

[80] Bélanger M., Mireille I., Magistretti P.J. (2011). Brain Energy Metabolism: Focus on Astrocyte-Neuron Metabolic Cooperation. Cell Metab..

[81] Cardanho-Ramos C., Morais V.A. (2021). Mitochondrial Biogenesis in Neurons: How and Where. Int. J. Mol. Sci..

[82] Chen B., Wang Q., Wang Y., Liu Q., Chen W., Mao H., Li J., Liu Q., Zhou X. (2025). Mitochondrial quality control in neurodegenerative diseases: From molecular mechanisms to natural product therapies. Front. Physiol..

[83] Coelho C., Fão L., Mota S., Rego A.C. (2022). Mitochondrial function and dynamics in neural stem cells and neurogenesis: Implications for neurodegenerative diseases. Ageing Res Rev..

[84] Sukhorukov V.S., Baranich T.I., Egorova A.V., Akateva A.V., Okulova K.M., Ryabova M.S., Skvortsova K.A., Dmitriev O.V., Mudzhiri N.M., Voronkov D.N. (2024). Mitochondrial Dynamics in Brain Cells During Normal and Pathological Aging. Int. J. Mol. Sci..

[85] Chan D.C. (2020). Mitochondrial Dynamics and Its Involvement in Disease. Annu. Rev. Pathol..

[86] Pickles S., Vigié P., Youle R.J. (2018). Mitophagy and Quality Control Mechanisms in Mitochondrial Maintenance. Curr. Biol..

[87] Westermann B. (2010). Mitochondrial fusion and fission in cell life and death. Nat. Rev. Mol. Cell Biol..

[88] Youle R.J., van der Bliek A.M. (2012). Mitochondrial fission, fusion, and stress. Science.

[89] Narendra D.P., Jin S.M., Tanaka A., Suen D.F., Gautier C.A., Shen J., Cookson M.R., Youle R.J. (2010). PINK1 is selectively stabilized on impaired mitochondria to activate Parkin. PLoS Biol..

[90] Lazarou M., Sliter D., Kane L., Sarraf S.A., Wang C., Burman J.L., Sideris D.P., Fogel A.I., Youle R.J. (2015). The ubiquitin kinase PINK1 recruits autophagy receptors to induce mitophagy. Nature.

[91] Twig G., Elorza A., Molina A.J., Mohamed H., Wikstrom J.D., Walzer G., Stiles L., Haigh S.E., Katz S., Las G. (2008). Fission and selective fusion govern mitochondrial segregation and elimination by autophagy. EMBO J..

[92] Deas E., Wood N.W., Plun-Favreau H. (2011). Mitophagy and Parkinson’s disease: The PINK1-parkin link. Biochim. Biophys. Acta.

[93] Meissner C., Lorenz H., Weihofen A., Selkoe D.J., Lemberg M.K. (2011). The mitochondrial intramembrane protease PARL cleaves human Pink1 to regulate Pink1 trafficking. J. Neurochem..

[94] Narendra D., Tanaka A., Suen D.F., Youle R.J. (2008). Parkin is recruited selectively to impaired mitochondria and promotes their autophagy. J. Cell Biol..

[95] Kondapalli C., Kazlauskaite A., Zhang N., Woodroof H.I., Campbell D.G., Gourlay R., Burchell L., Walden H., Macartney T.J., Deak M. (2012). PINK1 is activated by mitochondrial membrane potential depolarization and stimulates Parkin E3 ligase activity by phosphorylating Serine 65. Open Biol..

[96] Gegg M.E., Cooper J.M., Chau K.Y., Rojo M., Schapira A.H., Taanman J.W. (2010). Mitofusin 1 and mitofusin 2 are ubiquitinated in a PINK1/parkin-dependent manner upon induction of mitophagy. Hum. Mol. Genet..

[97] Heo S.J., Thorpe S., Driscoll T., Duncan R.L., Lee D.A., Mauck R.L. (2015). Biophysical Regulation of Chromatin Architecture Instills a Mechanical Memory in Mesenchymal Stem Cells. Sci. Rep..

[98] Yamano K., Matsuda N., Tanaka K. (2016). The ubiquitin signal and autophagy: An orchestrated dance leading to mitochondrial degradation. EMBO Rep..

[99] Quinn P.M.J., Moreira P.I., Ambrósio A.F., Alves C.H. (2020). PINK1/PARKIN signalling in neurodegeneration and neuroinflammation. Acta Neuropathol. Commun..

[100] Whitworth A.J., Pallanck L.J. (2017). PINK1/Parkin mitophagy and neurodegeneration-what do we really know in vivo?. Curr. Opin. Genet. Dev..

[101] Wasner K., Smajic S., Ghelfi J., Delcambre S., Prada-Medina C.A., Knappe E., Arena G., Mulica P., Agyeah G., Rakovic A. (2022). Parkin Deficiency Impairs Mitochondrial DNA Dynamics and Propagates Inflammation. Mov. Disord..

[102] Li J., Yang D., Li Z., Zhao M., Wang D., Sun Z., Wen P., Dai Y., Gou F., Ji Y. (2023). PINK1/Parkin-mediated mitophagy in neurodegenerative diseases. Ageing Res. Rev..

[103] Fang E.F., Hou Y., Palikaras K., Adriaanse B.A., Kerr J.S., Yang B., Lautrup S., Hasan-Olive M.M., Caponio D., Dan X. (2019). Mitophagy inhibits amyloid-β and tau pathology and reverses cognitive deficits in models of Alzheimer’s disease. Nat. Neurosci..

[104] West A., Khoury-Hanold W., Staron M., Tal M.C., Pineda C.M., Lang S.M., Bestwick M., Duguay B.A., Raimundo N., MacDuff D.A. (2015). Mitochondrial DNA stress primes the antiviral innate immune response. Nature.

[105] Mihaylova M.M., Shaw R.J. (2011). The AMPK signalling pathway coordinates cell growth, autophagy and metabolism. Nat. Cell Biol..

[106] Kim D.H. (2024). Contrasting views on the role of AMPK in autophagy. Bioessays.

[107] Trefts E., Shaw R.J. (2021). AMPK: Restoring metabolic homeostasis over space and time. Mol. Cell.

[108] Rezaeian A.H., Wei W., Inuzuka H. (2022). The regulation of neuronal autophagy and cell survival by MCL1 in Alzheimer’s disease. Acta Mater. Med..

[109] Kim J., Kundu M., Viollet B., Guan K.L. (2011). AMPK and mTOR regulate autophagy through direct phosphorylation of Ulk1. Nat. Cell Biol..

[110] Li R.L., Wu S.S., Wu Y., Wang X.X., Chen H.Y., Xin J.J., Li H., Lan J., Xue K.Y., Li X. (2018). Irisin alleviates pressure overload-induced cardiac hypertrophy by inducing protective autophagy via mTOR-independent activation of the AMPK-ULK1 pathway. J. Mol. Cell. Cardiol..

[111] Pickford F., Masliah E., Britschgi M., Lucin K., Narasimhan R., Jaeger P.A., Small S., Spencer B., Rockenstein E., Levine B. (2008). The autophagy-related protein beclin 1 shows reduced expression in early Alzheimer disease and regulates amyloid beta accumulation in mice. J. Clin. Investig..

[112] Lior N., Chen D., Dan F., Ronit P.K. (2025). The connection between autophagy and Alzheimer’s disease. Inflamm. Res..

[113] Fernandes S.M., Mayer J., Nilsson P., Shimozawa M. (2025). How close is autophagy-targeting therapy for Alzheimer’s disease to clinical use? A summary of autophagy modulators in clinical studies. Front. Cell Dev. Biol..

[114] Caccamo A., Majumder S., Richardson A., Strong R., Oddo S. (2010). Molecular interplay between mammalian target of rapamycin (mTOR), amyloid-beta, and Tau: Effects on cognitive impairments. J. Biol. Chem..

[115] Festa B.P., Barbosa A.D., Rob M., Rubinsztein D.C. (2021). The pleiotropic roles of autophagy in Alzheimer’s disease: From pathophysiology to therapy. Curr. Opin. Pharmacol..

[116] Swerdlow R.H., Burnsk J.M., Khank S.M. (2014). The Alzheimer’s disease mitochondrial cascade hypothesis: Progress and perspectives. Biochim. Biophys. Acta.

[117] Goenawan H., Kiasati S., Sylviana N., Megantara I., Lesmana R. (2023). Exercise-Induced Autophagy Ameliorates Motor Symptoms Progressivity in Parkinson’s Disease Through Alpha-Synuclein Degradation: A Review. Neuropsychiatr. Dis. Treat..

[118] Mizushima N., Komatsu M. (2011). Autophagy: Renovation of cells and tissues. Cell.

[119] Johansen T., Lamark T. (2020). Selective Autophagy: ATG8 Family Proteins, LIR Motifs and Cargo Receptors. J. Mol. Biol..

[120] Hara T., Nakamura K., Matsui M., Yamamoto A., Nakahara Y., Suzuki-Migishima R., Yokoyama M., Mishima K., Saito I., Okano H. (2006). Suppression of basal autophagy in neural cells causes neurodegenerative disease in mice. Nature.

[121] Wallace D.C. (2005). A mitochondrial paradigm of metabolic and degenerative diseases, aging, and cancer: A dawn for evolutionary medicine. Annu. Rev. Genet..

[122] Ureshino R.P., Erustes A.G., Bassani T.B., Wachilewski P., Guarache G.C., Nascimento A.C., Costa A.J., Smaili S.S., da Silva Pereira G.J. (2019). The Interplay between Ca2+ Signaling Pathways and Neurodegeneration. Int. J. Mol. Sci..

[123] Murphy M.P. (2009). How mitochondria produce reactive oxygen species. Biochem. J..

[124] Atlante A., Valenti D. (2023). Mitochondrial Complex I and β-Amyloid Peptide Interplay in Alzheimer’s Disease: A Critical Review of New and Old Little Regarded Findings. Int. J. Mol. Sci..

[125] Pohland M., Pellowska M., Asseburg H., Hagl S., Reutzel M., Joppe A., Berressem D., Eckert S.H., Wurglics M., Schubert-Zsilavecz M. (2018). MH84 improves mitochondrial dysfunction in a mouse model of early Alzheimer’s disease. Alzheimer’s Res. Ther..

[126] D’Alessandro M.C.B., Kanaan S., Geller M., Praticò D., Daher J.P.L. (2025). Mitochondrial dysfunction in Alzheimer’s disease. Ageing Res. Rev..

[127] Exner N., Lutz A.K., Haass C., Winklhofer K.F. (2012). Mitochondrial dysfunction in Parkinson’s disease: Molecular mechanisms and pathophysiological consequences. EMBO J..

[128] Berridge M.J. (2014). Calcium regulation of neural rhythms, memory and Alzheimer’s disease. J. Physiol..

[129] Nunnari J., Suomalainen A. (2012). Mitochondria: In sickness and in health. Cell.

[130] Pickrell A.M., Youle R.J. (2015). The roles of PINK1, parkin, and mitochondrial fidelity in Parkinson’s disease. Neuron.

[131] Misrani A., Tabassum S., Yang L. (2021). Mitochondrial Dysfunction and Oxidative Stress in Alzheimer’s Disease. Front. Aging Neurosci..

[132] Tran M., Reddy P.H. (2021). Defective Autophagy and Mitophagy in Aging and Alzheimer’s Disease. Front. Neurosci..

[133] Laurindo L.F., Dogani Rodrigues V., Fornari Laurindo L., Amaral Cherain L.M., Pereira de Lima E., Leme Boaro B., da Silva Camarinha Oliveira J., Chagas E.F.B., Catharin V.C.S., Haber J.F.D.S. (2025). Targeting AMPK with Irisin: Implications for metabolic disorders, cardiovascular health, and inflammatory conditions—A systematic review. Life Sci..

[134] Zhang X., Xu S., Hu Y., Liu Q., Liu C., Chai H., Luo Y., Jin L., Li S. (2023). Irisin exhibits neuroprotection by preventing mitochondrial damage in Parkinson’s disease. NPJ Park. Dis..

[135] Qiu R., Sun W., Su Y., Sun Z., Fan K., Liang Y., Lin X., Zhang Y. (2024). Irisin’s emerging role in Parkinson’s disease research: A review from molecular mechanisms to therapeutic prospects. Life Sci..

[136] Chen W., Zhao H., Li Y. (2023). Mitochondrial dynamics in health and disease: Mechanisms and potential targets. Signal Transduct. Target. Ther..

[137] Grel H., Woznica D., Ratajczak K., Kalwarczyk E., Anchimowicz J., Switlik W., Olejnik P., Zielonka P., Stobiecka M., Jakiela S. (2023). Mitochondrial Dynamics in Neurodegenerative Diseases: Unraveling the Role of Fusion and Fission Processes. Int. J. Mol. Sci..

[138] Jodeiri Farshbaf M., Alviña K. (2021). Multiple Roles in Neuroprotection for the Exercise Derived Myokine Irisin. Front. Aging Neurosci..

[139] Ma M., Jing G., Tian Y., Yin R., Zhang M. (2025). Irisin: Emerging Therapeutic Targets for Cognitive Impairment-Related Diseases. Expert. Rev. Mol. Med..

[140] Tu T., Yin S., Pang J., Zhang X., Zhang L., Zhang Y., Xie Y., Guo K., Chen L., Peng J. (2021). Irisin Contributes to Neuroprotection by Promoting Mitochondrial Biogenesis After Experimental Subarachnoid Hemorrhage. Front. Aging Neurosci..

[141] Mary A., Eysert F., Checler F., Chami M. (2023). Mitophagy in Alzheimer’s disease: Molecular defects and therapeutic approaches. Mol. Psychiatry.

[142] Flori L., Testai L., Calderone V. (2021). The “irisin system”: From biological roles to pharmacological and nutraceutical perspectives. Life Sci..

[143] Mohammad Rahimi G.R., Hejazi K., Hofmeister M. (2022). The effect of exercise interventions on Irisin level: A systematic review and meta-analysis of randomized controlled trials. EXCLI J..

[144] Minuti A., Raffaele I., Scuruchi M., Lui M., Muscarà C., Calabrò M. (2025). Role and Functions of Irisin: A Perspective on Recent Developments and Neurodegenerative Diseases. Antioxidants.

[145] Lee K., Kim M. (2025). Evolutionary Insights into Irisin/FNDC5: Roles in Aging and Disease from Drosophila to Mammals. Biomolecules.

[146] Han C., Zhou Z., Kong L., Lu J., Li X. (2025). Association Between Irisin Level and Cognitive Function: A Systematic Review and Meta-Analysis. Brain Behav..

[147] Shi X., Gu Q., Fu C., Ma J., Li D., Zheng J., Chen S., She Z., Qi X., Li X. (2024). Relationship of irisin with disease severity and dopamine uptake in Parkinson’s disease patients. Neuroimage Clin..

[148] Jedrychowski M.P., Wrann C.D., Paulo J.A., Gerber K.K., Szpyt J., Robinson M.M., Nair K.S., Gygi S.P., Spiegelman B.M. (2015). Detection and Quantitation of Circulating Human Irisin by Tandem Mass Spectrometry. Cell Metab..

